# Secure data sharing with blockchain for remote health monitoring applications: a review

**DOI:** 10.1007/s40860-023-00204-w

**Published:** 2023-05-11

**Authors:** Venkatesh Upadrista, Sajid Nazir, Huaglory Tianfield

**Affiliations:** Department of Computing, Glasgow Caledonian University, Glasgow, G4 0BA Scotland

**Keywords:** Security and privacy, Scalability, Reliability, Single-point-of-failure, Latency

## Abstract

Remote Health Monitoring (RHM) is going to reinvent the future healthcare industry and bring about abundant value to hospitals, doctors, and patients by overcoming the many challenges currently being faced in monitoring patient’s well-being, promoting preventive care, and managing the quality of drugs and equipment. Despite the many benefits of RHM, it is yet to be widely deployed due to the healthcare data security and privacy challenges. Healthcare data are highly sensitive and require fail-safe measures against unauthorized data access, leakages, and manipulations, and as such, there are stringent regulations governing how healthcare data can be secured, communicated, and stored, such as General Data Protection Regulation (GDPR) and the Health Insurance Portability and Accountability Act (HIPAA). The challenges and regulatory demands in RHM applications can be addressed using blockchain technology due to its distinguishing features of decentralization, immutability, and transparency to address the challenges of data security and privacy. This article will provide a systematic review on the use of blockchain in RHM, focusing primarily on data security and privacy.

## Introduction

Remote healthcare is currently gaining lot of attention as it can help to increase the overall lifespan of individuals and ensure a better quality of life. The healthcare sector can be improved by remotely monitoring health indicators through portable sensing devices [1] and via the introduction of the latest communications and innovative digital technologies. These advanced digital technologies can assist doctors and medical practitioners in the early diagnosis of various diseases and can help physicians to remotely monitor patients’ health, thereby reducing the onsite load on doctors and nurses [1, 2]. In recent years, there has been a growing interest in creating effective and flexible healthcare arrangements via the remote monitoring of patients, in particular those who are deemed as vulnerable [3]. The average spending on smart healthcare in Europe is approximately 10% of gross domestic product (GDP), and approximately 99 billion euros of healthcare costs were projected to be saved through smart healthcare by 2020 [4]. RHM covers a range of solutions, such as delivering high-quality acute care at home through continuous monitoring and timely alerts to doctors and nurses. It also allows patients to recover from the comfort of their own home with transitional care programs that support early discharge or admission avoidance, as well as provide support with chronic care that can help patients better manage their conditions, delivered at home with a personalized experience. RHM can not only enable better patient-doctor performance, but at the same time, it can reinvent the health sector with innovative use cases enabling predictive healthcare in contrast to reactive approaches. For example, impending heart attacks or cancer progression could be detected, potentially saving millions of lives. Additionally, one of the other significant benefits of applying Internet of Medical Things (IoMT) to healthcare is the lower overall cost of care.

With the right IoMT setup in place, the health sector stands to save tens of billions of dollars every year. Federal Communications Commission (FCC) drafted by the National Broadband Plan for example stated that using RHM technologies along with electronic health records (EHR) could save the healthcare industry $700 billion over the next 2 decades [5]. RHM is one of the most important focus areas for the health care sector as many countries including the UK are suffering from a dramatic increase in the number of patients, making it increasingly difficult for them to access doctors or other primary caregivers.

Although the Internet of Things (IoT) can be applied in multiple industries, the technology brings immense value to the health sector as it can bring a change to the lives and health of individuals [3]. There are numerous sensors that can be used to monitor activity as well as record health information. Wearable devices like smart bands, smartwatches, or non-wearable devices like smartphones or glucose-level monitors can continuously monitor, collect, and record data from the human body [1]. There are already a wide variety of reliable and wearable devices for healthcare monitoring on the market [6] which can be used to enhance the RHM for preventive care [2]. While monitoring devices helps in the treatment of patients, it can also allow doctors to observe patients’ conditions remotely, leading to early prevention [2] and intervention for different diseases and types of attacks [7, 8].

**Fig. 1:**
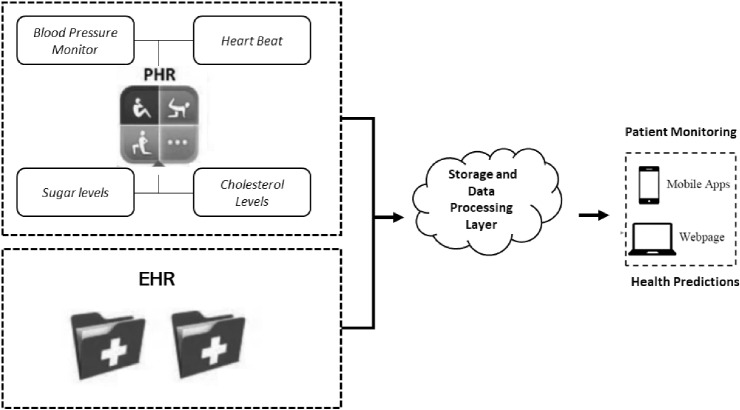
Health data from multiple sources can be combined for patient monitoring and disease predictions

As depicted in Fig. 1, transactional health data, such as heartbeats, temperature, blood pressure, glucose levels, cholesterol, and sugar levels combined with electronic health records data, can predict when a person is in danger of an impending disease. For example, a patient with heart issues can have their blood pressure and cholesterol levels monitored at regular intervals using IoMT-enabled wearable devices. Combining data from EHR with any abnormal blood pressure and cholesterol levels can send alerts to a doctor in case of a developing risk of another heart attack.

A widespread adoption of RHM systems is, however, often hindered by challenges from a security and data privacy perspective [9]. Some monitoring applications are required to store and process patients’ data at device level (i.e., the edge), the cloud or potentially a combination of both, and such a distributed nature of IoT systems introduces new security and privacy challenges [10, 11]. The migration of large-scale computing and storage services to the edge creates exposure to attacks, and therefore, there is a requirement to control the network or prevent attacks on the edge resources [12, 13]. On the other hand, there could be significant ambiguity regarding data ownership and regulation, which indicates that data generated by IoMT are prone to theft and makes the data susceptible to cybercriminals that can hack into the system to compromise personal health information [14]. Similarly, centralized cloud architectures can be a single-point-of-failure [15] which can potentially disrupt the entire network [16, 17]. Moreover, patient-related data storage on a cloud can add communication overhead and delays for data retrieval, although this does require less data management effort [33]. It is also not straightforward to implement secure data sharing in healthcare industry where there is often a lack of transparency and trust among participants [18]. Attackers can hack health data without users’ permission [3], leading to a leakage of sensitive patient information. The feasibility and implementation of a hybrid approach of data offloading and data sharing for healthcare applications remains unsolved [16, 19–22], which urgently requires further innovative solutions. Specifically, the EHR concept provides a way to store a huge amount of sensitive medical data, but interoperability can be a challenge among health centers due to privacy concerns [22, 23].

There are compelling reasons and associated benefits of applying IoT to RHM; however, as discussed above, the technology still has several limitations. Several proposals discuss the impact of attacks on remote monitoring healthcare systems with catastrophic results [2, 3, 24]. On the other hand, healthcare data need to be made available to doctors and patients on a real-time basis, as any delays during critical care situations can be fatal. This is where blockchain technology can help overcome the data security and privacy challenges of using IoT in healthcare and its associated benefits for RHM [25]. The major reason for using the blockchain in RHM is its prominent features to enhance security and privacy of patients’ data [23] and securely aggregating this with EHR data [22]. Multiple blockchain infrastructures have emerged, with the most prominent ones being public private blockchains. Public blockchains can be permissionless or permissioned, whereas private blockchains are always permissioned. The two widely known blockchain-based approaches are Ethereum and Hyperledger Fabric framework [26]. Ethereum is a public network that enables permissioned or permissionless networks [26, 27] and Hyperledger is a permissioned private network designed for operations involving confidential and sensitive data [26].

Integrating blockchain with RHM platforms is considered a solution to data security and privacy challenges [10, 11, 25]. Blockchain can harness the data stream to improve the quality of remote care provided by streamlining the sharing of medical records [23], protecting sensitive data from hackers, and give patients more control over their information. The intrinsic nature of blockchain brings several unique features, such as decentralization, transparency, and traceability, meaning that it can potentially address the aforementioned security and privacy challenges [17, 28]. Since blockchain has been widely used to address security issues in distributed scenarios, there are many approaches to enhance the privacy and security problems by replacing existing security models with blockchain [29, 30]. In addition, the combination of IoT and blockchain with deep-learning models can enhance security postures. Deep learning is commonly used in vision tasks, such as semantic segmentation, image captioning, object detection, recognition, and image classification [31] that can benefit RHM use cases while dealing with clinical images.

Although blockchain promises many benefits, there are also several challenges concerning areas of security and privacy, latency, and reliability. Some reviews have focused on security and privacy [7, 32–34], whereas others have considered latency challenges [35, 36], but often these challenges are not discussed together. In contrast, we have provided a comprehensive review on the state-of-art of blockchain for RHM, with a focus on security, privacy, latency, and reliability.

## RHM using blockchain technology: system architecture

With recent technology developments, the concept of remote monitoring has come to the forefront [1–3]. Remote monitoring uses a new generation of information technologies, such as IoMT and cloud computing, to transform the traditional healthcare system, making it efficient, convenient, and personalized [37]. Remote monitoring not only brings abundant value to hospitals, doctors, and patients by overcoming many challenges currently faced in monitoring individual’s well-being and promoting preventive care, but has also proven effective in tackling the most pressing challenges in the healthcare sector such as preventive care for life-threatening diseases.

### System components

Remote monitoring consists of multiple participants, including doctors, patients, hospitals, and even research institutions. It also has multiple dimensions, such as health monitoring, early disease diagnosis, and treatment. IoMT, cloud computing, and blockchain together with modern biotechnology constitute the cornerstone of remote monitoring, and these technologies are widely used in all aspects of remote monitoring. To understand what an IoMT architecture is for RHM, it is essential to understand what goes into a complete IoMT system.

First, an IoMT system needs hardware—sensors or devices [38] such as wearable devices or devices where patients can send their health data. These sensors and devices collect data from patients or perform actions, such as informing doctor or nurses when the blood pressure of a patient exceeds a certain threshold.

An IoMT system also needs connectivity [38]. The hardware (device) needs a way to transmit all that data to the cloud (e.g., sending patient data) or needs a way to receive commands from the cloud (e.g., send a SMS to doctor). This can be accomplished with mature forms of connectivity like cellular, satellite, or Wi-Fi, or may necessitate more recent, IoMT-focused connectivity options like LoRaWAN.

Cloud is another important element in the architecture where IoMT software is hosted [39]. This software on the cloud is responsible for storing and analyzing the data collected from the sensors and then making decisions. As an example, blood pressure readings are stored on the cloud, and if an abnormal reading is detected, actions are taken such as sending a message to a doctor.

Finally, an IoMT system needs a user interface. To make all this useful, there needs to be a way for users to interact with the IoT system; for example, this could be a web-based app with a dashboard that shows all readings of a patient such as blood pressure, cholesterol levels, blood sugar levels, etc., and allow doctors to prescribe treatment.

Security is another important element in an IoMT context [16, 19, 20, 32], since sensitive data are exchanged between different entities over wired or wireless networks. Data from IoMT devices then need to be securely sent to the cloud or edge for analysis and decisions.

**Fig. 2:**
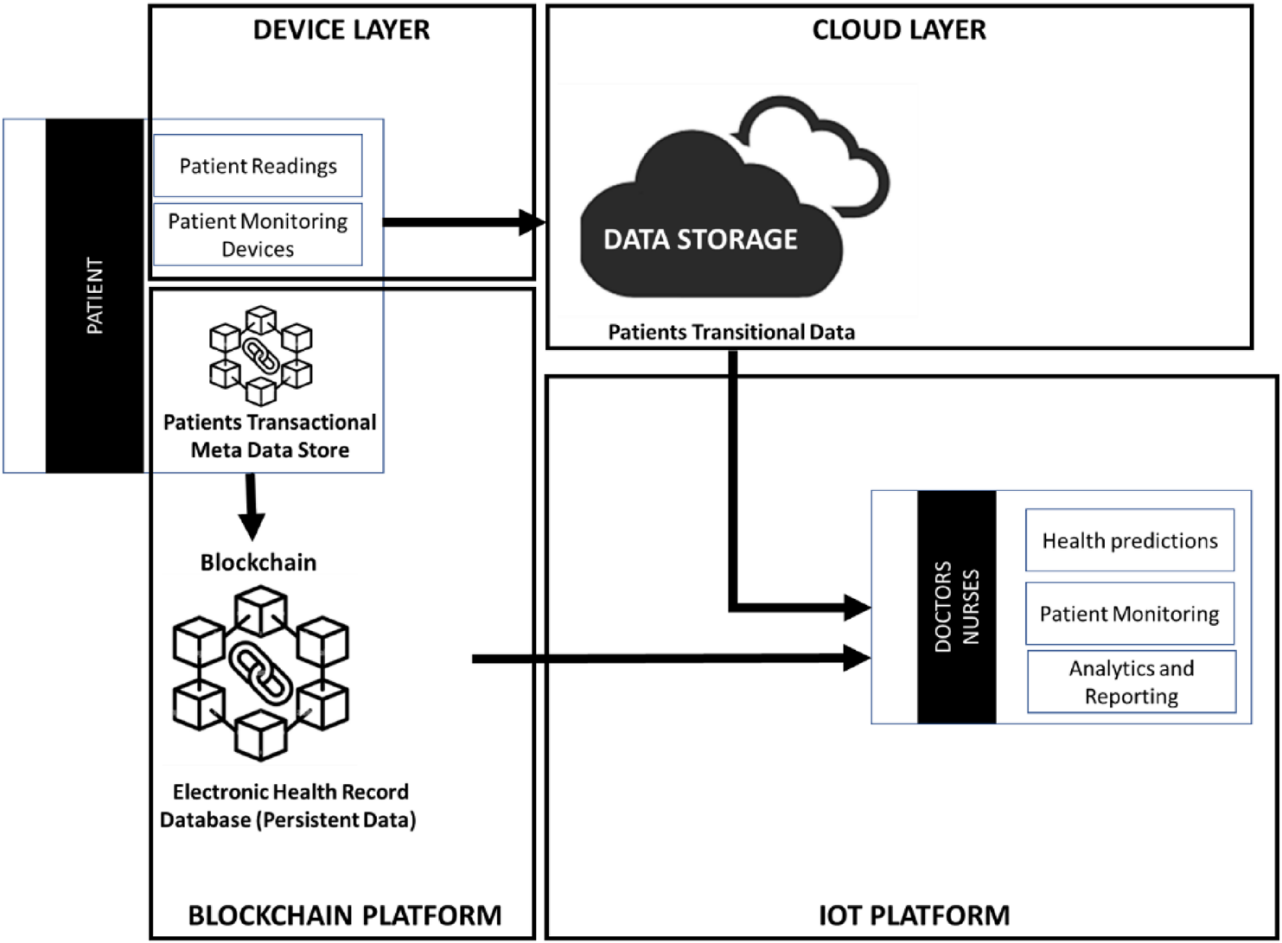
Blockchain-based RHM architecture

The above steps translated to an IoMT architecture is depicted in Fig. 2, which is appropriate for most RHM use cases.

### Device layer (the sensors and actuators)

As the basis for every IoMT system, connected healthcare devices or connected objects are responsible for providing data which are effectively the essence of the IoMT. To pick up parameters from the outside world, such as patients’ health data, devices need sensors, fundamentally RHM devices are composed of both software and hardware. Some modern devices also have an operating system, a computer, storage, and connectivity. Some examples of IoMT devices are blood pressure monitors, glucometers, pulse oximeters, telemetry device, and activity trackers, which track patient’s steps, heart rate, fall risk, and even sleep. Other devices figure prominently in remote wellness and chronic disease monitoring, such as infusion and insulin pumps.

### Cloud platform

RHM devices attached to a patient’s body send data to the cloud. These devices are connected to the cloud through a variety of methods including cellular, satellite, Wi-Fi, Bluetooth, low-power wide-area networks (LPWAN) or connected directly to the internet via the Ethernet [40, 41].

The IoMT cloud platform is designed to store, process, and analyze large volumes of data for deeper insights using powerful data analytics engines and machine learning mechanisms. It is a platform to create and manage applications, run analytics, and store and secure patient’s health data, as well as create reports that end users such as doctors and nurses can view to make a decision or perform an action.

Once the data are sent to the cloud, software on the IoT cloud platform performs data processing—this could be very simple data processing task such as checking that the heart rate reading of a patient is within an acceptable range. Alternatively, this could be a very complex task, such as combining blood pressure, body temperature, electrocardiogram, and echocardiogram data of the patient to determine if a certain threshold is reached upon which the chance of a heart attack can be estimated. Based on the data processed, the next step performed by the IoMT cloud platform is alerting and notifying. This could for example be an alert to the doctor or nurse (via email, text, or call notification) when the temperature is too high for the patient.

Depending on the IoMT use case, the user may also be able to perform an action based on a certain condition. For example, a doctor may decide to start monitoring the patients’ blood pressure every hour if the temperature of the patient crosses a specific limit. An IoT platform can also manage devices (the sensors and actuators) and there are multiple IoT cloud platforms that exist in the market today and most of these platforms support device management.

### Blockchain

Data exchange between the devices and the cloud needs to be accurate and fully secured. In the context of RHM use cases, sensitive patient medical data are stored and exchanged between devices and the cloud, and therefore, it is imperative that the right security mechanisms are in place to ensure these data are secured. Blockchain is one of the technologies that can provide enormous value to RHM use cases from a security and privacy perspective [23]. A blockchain is “a distributed database that maintains a continuously growing list of ordered records, called blocks.” These blocks are linked using cryptography and each block contains a cryptographic hash of the previous one, as well as a timestamp and transactional data. These blocks are then stored on nodes which are comparable to small servers. On a blockchain, all the nodes are connected to each other known as a peer-to-peer (P2P) network, and they continuously exchange the newest information on the blockchain with each other which ensures that all nodes are always updated. The nodes store a complete copy of the distributed ledger and are responsible for the reliability of the stored data. There are two types of nodes in blockchain. One is known as the full node [42] which contains a copy of the blockchain's history, including all blocks created, and second is the light node [42] which only downloads the essential data from processed transactions. In a public blockchain, anyone can join the network and become a node by synchronizing their system with the blockchain data, whereas in a private blockchain, participants can join only through an invitation [43, 44] where their identity or other required information must be verified.

RHM systems can utilize blockchain-based smart contracts to secure data as part of the architecture. With smart contracts, only authorized viewers can read the block and designated nodes can verify new blocks within the system. Security can be imposed by introducing the concept of valid blocks that must contain signatures from a minimum number of members. No rogue nodes can then insert false transactions into the chain as only pre-authorized verified nodes can be a member of the network. This architecture mainly exploits the advantages of the smart contract of the blockchain system. Smart contracts are modular and can be customized to send notifications to patients and medical professionals.

#### Smart contracts

Smart contracts are programs stored on a blockchain that run when predetermined conditions are met [45] by following simple “if/when…then…” statements that are written into the code on the blockchain. A network of computers executes the actions when predetermined conditions have been met and verified, for example,

***If body temperature of Patient1 is more than 120 °C, then send an SMS to Doctor1.***

**Fig. 3:**
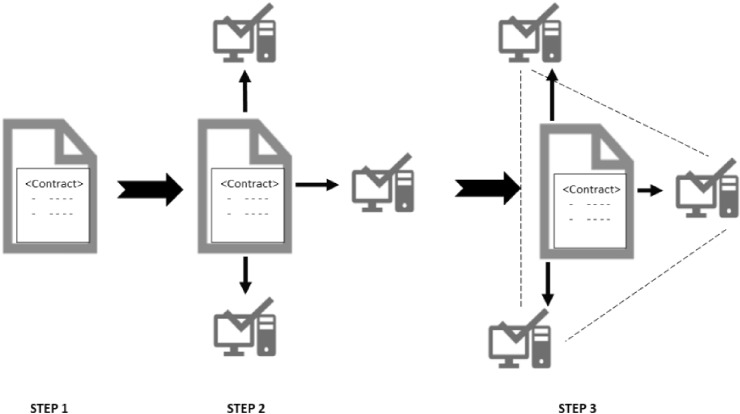
Smart contract workflow

A smart contract first needs to be signed by all participants who are part of the blockchain network, and once everyone signs the contract, it is attached to the blockchain and broadcast on the entire network. Every smart contract has its address in the blockchain upon which participants can interact with it. The application flow of the smart contracts is depicted in Fig. 3.

The first step in the process is to manually agree on the terms and conditions of the contract and then transfer these into a smart contract by translating it using a programming code, e.g., solidity programming language. The code exactly represents the conditional statements that describe the possible scenarios for a future transaction.

Once the code is created, the second step is for the smart contract to be stored on the blockchain network and replicated among all participants.

Finally in the third step, the code is run and executed by all nodes in the network. If a term of the contract is satisfied and it is verified by all participants of the blockchain network, then the relevant transaction is executed.

## Remote health monitoring systems

Remote healthcare is an emerging research field as the world moves toward remote monitoring, real time, and fast detection of illnesses [2]. Remote healthcare has many categories including telehealth [7, 8] and mobile health monitoring all of which involve the monitoring of patients outside the hospital environment through technology. RHM technologies have been identified as a viable alternative to improve access to care which has already proven its importance, most notably during the COVID-19 pandemic [46].

Telehealth and telemedicine are two terms broadly used in connection with RHM. The former deals with various digital health services, such as remote monitoring, video visits, and online written communication, while the latter concerns patient engagement with doctors using a combination of video or phone and remote monitoring devices. Using these technologies, patients in remote and underdeveloped areas can access healthcare services that may not otherwise receive without traveling a great distance or overcoming other logistical barriers [8, 47].

A large number of successful RHM platforms are designed and developed for seamless integration into healthcare provider networks to increase adoption and offer remote care. As part of this review, we try to elicit these perspectives from a wide range of papers in the adoption and integration of RHM into clinical workflows. Table 1 provides a summary.

**Table 1:** Comparative study for RHM

Ref #	Implementation platform	Edge computing	Data storage	Health monitoring devices used	Visualization tools
[48]	Arduino Yun board	No	Cloud	Heart rate and blood oxidation level (SpO2)	Unspecified
[47]	Unspecified	Yes	Cloud	Blood pressure, temperature, heart rate, respiratory rate, sleep and stress level devices	Smart devices, laptops and computers
[49]	MATLAB tools / Android	No	Files / Folders	Monitoring of ECG	Mobile (Android)
[7]	RESTful Web Service Sensor and JSON/ arrays for ECG, SpO2	No	Cloud	Monitoring of ECG, SpO2, temperature and physical movements	Mobile devices and tablets
[8]	JavaScript and JSON files,	No	Local database	ECG Device	Webpages, Mobile apps

Authors in ref [48] proposed a prototype WLAN-based RHM system with an end-to-end flow. The system was developed to collect the patient’s crucial signals, such as blood pressure, pulse oximetry, body temperature, and heart rate, using medical sensors. Arduino Yun board was used as the main controller for processing and analyzing the patient data which was uploaded to the cloud using the Application Programming Interface (API) via WLAN. Doctors were provided with an interface to log into the online server for checking the patient’s medical data and to send their commentary to the patient through the server. In case of a loss of WLAN connection, the data were temporarily stored, to be synchronized with the server once the connection was restored. An alert was sent automatically by the system in case of emergencies.

In [47], authors discussed a smart health-monitoring system based on fog nodes. In this system, edge users were equipped with various kinds of wearable sensors and devices [1] to aggregate medical measurements. Low-power wide-area network (LoRaWAN) protocol was used to send aggregated measurements directly to the nearest fog nodes or health centers. The use of the LoRaWAN gateway made it possible to transmit the data recorded by those devices to the primary health centers, tens of kilometers away even without an Internet facility. This can be a huge benefit in remote places where there is an absence of a reliable Internet connection or any Internet facilities. The data sent through these devices could be interconnected with the smart devices, laptops, or computers of the medical personnel and backed up on the health center’s storage. The proposed architecture can be used to solve the resource constraints of clinic-centric health systems with a smart technology solution.

A telemedicine application constitutes of both hardware and software components at both the patient and doctor ends. The field of cardiology where ECG is the major tool for diagnosis is one of the leading fields for the application of telemedicine. An image-based technique to acquire and analyze constant streaming of ECG signal used MATLAB tools and a data communication system based on an Internet network [49]. Vital signs and parameters, such as heart rate [from the Intensive Care Unit (ICU) monitoring machine], were captured using a webcam and the image was then transmitted through the Internet. Any anomalies were sent as a notification to the doctor’s phone and data were stored on folders in a centralized server accessible to all doctors. The mobile phone stored the recorded data in an on-device SQLite database temporarily and was also forwarded to the server in real time.

Usually, after a patient is moved to the ward, their physiological parameters are not continuously monitored, which can lead to a relapse. A low-cost modular monitoring system prototype was built for mobile support to facilitate faster and better medical interventions for patients discharged from an ICU to a ward for further evaluation [7]. The system was developed using low-power dedicated sensor arrays for monitoring electrocardiogram (ECG), blood oxidation level (SpO2), temperature, and movement. A RESTful web interface was developed to provide platform agnostic behavior and the flexibility to integrate new components. The sensor blocks were linked to a central control unit. Low-power radio interfaces (i.e., IEEE 802.15.4) were used to enable communication between the sensors and the control unit. The system integrated with mobile devices for real-time data visualization, and a degree of freedom for non-critical patients. The system could be especially useful when medical staff are limited to supporting only emergency cases. It could also be extended to allow for chronic patient remote monitoring (such as people suffering from asthma and chronic obstructive disease), with adequate security added to the system.

Authors in [8] discussed the importance of RHM for people in remote rural areas to obtain professional healthcare services. They proposed an IoT-based real-time RHM system that guaranteed the integrity of the real-time electrocardiogram (ECG). The system supported both real-time and store-and-forward modes. The advantage of a store-and-forward mode is that it has no data integrity issues, and additionally, the model does not require a high network quality. The real-time node did, however, become challenging, since packet loss and transmission delays during real-time transmissions can affect the data integrity, especially for ECG signal transmission. This meant that inaccurate health data might could have led to a misdiagnosis. Message Queuing Telemetry Transport (MQTT) protocol was used to transmit the ECG data in real time from the proposed system to the webserver. Smartphones or a computer can be then used by doctors to access the webserver and obtain real-time or previously recorded ECG data.

## Security and privacy challenges in RHM systems

In this section, we discuss the security and privacy challenges faced by RHM systems. This can include attacks against the IoMT device, exploiting vulnerabilities in the encryption algorithms and communications protocols, or unauthorized access to data. The literature included in this section was aimed at addressing the security and privacy challenges but have not considered blockchain as one of the design elements. A summary of the healthcare data used and the security techniques for the various systems is provided in Table 2. Moreover, we discuss the main challenges considered in the reviewed work and potential solutions that were adopted to ensure the security and safety of RHM systems.

**Table 2:** Comparative data of RHM addressing security and privacy

Ref	Implementation platform	Data store	Cryptographic model	Health monitoring devices used	Visualization tools
[50]	Unspecified	Cloud	Asymmetric keys / DC	BP device	Desktops and tools such as PowerBI
[9]	MIRACL libraries and	Cloud	Rabin authentication	Unspecified	Unspecified
[51]	Proprietary libraries and image recognition tools	Cloud	RC5, 2-D maps & 128-bit encryption	ECG, EEG, BP, SpO2, breath sensors imaging systems	Mobile devices
[52]	Holochain Platform	Holochain	DHT and DS	Patient position sensors, BP, pulse sensor, oxygen and temperature sensor	Smart apps (desktop and android)
[14]	Java applet & G&D smart card as monitoring platform	Local storage	Unspecified cryptographic functions	All health-monitoring units (HMUs)	Mobile devices
[46]	Custom built threat analysis framework (SHChecker)	Local storage	Proprietary threat analysis framework	BP, ECG, systolic, diastolic, nasal sound, SpO2, EEG	Raw output Messages such as text message
[53]	Markov Chain & Common Vulnerability Scoring	Cloud	Unspecified	Unspecified	Unspecified
[54]	Hidden Markov Model (HMM)	Cloud	None	Oximeter, smart scale and motion sensors	Mobile and desktop apps

### Communications protocols

Using a Microsoft threat modeling tool, current threats and vulnerabilities in IEEE 11073 protocol were identified [50]. Current RHM devices have a limitation on the number of people who can share a single device, and therefore, the use of near field communication (NFC) for identification was proposed in RHM devices for multi-user environments. This allowed multiple people to share a single device to reduce errors associated with incorrect user identification [55]. The proposed RHM security model will mitigate the security vulnerabilities and threat currently present in the RHM ecosystem as well as improve security.

Some of the most challenging security issues in the existing authentication protocols for RHM were described, and a lightweight public-key-based authentication protocol for Medical Sensor Networks (MSN) was proposed [9]. MSN can have limitations in computational power, data/energy storage, and communication range/bandwidth, and to overcome these limitations, MSNs have often been integrated with cloud computing. A modified version of the Rabin authentication algorithm was proposed to improve the signature signing process, thus making it suitable for delay-sensitive MSN applications. To prove the efficiency of the Rabin algorithm, it was implemented with different hardware settings using Tmote Sky motes and was also implemented on a Field Programmable Gate Arrays (FPGA) to evaluate its design and performance. Furthermore, the proposed protocol was implemented and tested using the Multi-precision Integer and Rational Arithmetic C/C +  + (MIRACL) library. The results showed that secure, direct, instant, and authenticated commands can be delivered from the medical staff to the MSN nodes, thereby making the architecture fool-proof.

### Use of cryptographic algorithms

On-body wireless networks (oBWNs) play a crucial role in improving healthcare services, and as such, security concerns with oBWNs have been investigated [47]. Using oBWNs, authorized users, such as health professionals or doctors, can access vital physiological information for a patient from wearable sensor nodes. Secure communication has always been the critical issue in oBWNs-based systems, due to the open nature of wireless communication and the sensitivity of patient information. A malicious adversary can easily intercept, modify, insert, and delete transmitted messages over insecure public communication channels [20]. In addition, it could be dangerous to the life of the patient if an unauthorized user gains access to the system, and sends instructions, for example, to stop the functioning of the wearable devices. The sensitivity of the physiological data collected by wearable sensor nodes is also a significant privacy concern in oBWNs. To overcome these challenges, a lightweight and secure three-factor authentication scheme for RHM using oBWNs was proposed. The proposed scheme used onetime hash chain techniques to enable forward secrecy and the pseudonym identity method was applied to provide user anonymity and resist against any desynchronization attack. The security analysis demonstrated that the proposed scheme provided superior security and functional features compared to previous iterations.

To ensure the security of patient’s critical data for RHM, authors in [51] proposed using the Rivest Cipher (RC5) algorithm. RC5 is a well-known cipher in terms of efficiency and overall performance [56, 57] and is suitable for a resource-constrained device in sensor-based applications due to its very low power, reduced memory usage, and easy adaptability. However, RC5 can be broken by attackers due to its poor diffusion property in key computation [58, 59]. To overcome this challenge, the authors proposed using RC5 in combination with chaos to enhance the security level, whereby chaos sequences can be used for the ciphering of medical images. With the increasing importance of medical images in medical diagnosis, the processing of the medical image becomes more intensive. Features, such as confusion and diffusion, make chaos-based cryptographic systems highly secure and reliable. 2-D chaotic logistic maps are much more complex when compared to the 1-D chaotic maps and represent a highly non-linear and complex dynamic system. The performance analysis of the proposed cryptosystem was carried out both visually and numerically. The statistical and experimental analysis clearly showed that the proposed scheme was very secure due to its large key space, strong resistance to different types of attacks, and extreme sensitivity to the cipher key and plain images.

### Data security

There is huge quantity of personal and clinical data that are generated by RHM devices [16]. Because of such data explosion, data security and data privacy become a major challenge [9, 10, 13, 24]. Although blockchain can be a potential solution to overcome this challenge, due to its inherent distributed ledger technology, it can suffer from major setbacks of increasing storage and computation requirements with the network size [26]. A holochain-based security and privacy-preserving framework for IoT was developed in [52] that can overcome these challenges and is particularly suited for resource-constrained IoT scenarios. Holochain is an emerging technology that provides an open-source distributed network infrastructure to communicate securely without inheriting the huge storage and data exchange [60]. The authors claimed that the proposed holochain framework facilitates completely distributed IoT-based smart healthcare systems, where holochain is implemented for storing information and ensuring its security and privacy. Holochain can be implemented at the network edge (i.e., on IoT nodes or fog nodes) as well as on the cloud. Cloud was proposed to process and store the transaction of a holochain network, since IoT devices are resource-constrained with limited memory, power, computation capacity, and energy [61]. In addition, holochain was demonstrated to offer high scalability, low processing costs, and low energy consumption making it ideal for realistic deployment for large-scale IoT RHM systems.

### Threats to IoT devices

Wearable and implantable medical sensors present many opportunities for providing timely health information for doctors and patients, but concerns about privacy and information quality do, however, impede the development and deployment of these technologies for RHM. A novel health-monitoring architecture was defined in [14] to enable security in addressing the weaknesses of commonly used personal health devices. The model used patient’s mobiles as a platform and an architecture was developed separating the personal device from the monitoring component. The architecture assumed that the health provider distributed only small-to-medium health-monitoring units (HMUs) to patients and the monitoring unit is always plugged into the device through a common interface such as an SD card, mini-USB, or SIM card. Although not all current phones have expansion slots, and GSM phones only have one SIM-card interface, authors considered next-generation mobile phones that have a standard expansion slot for this prototype. The HMU stores secret keys and can perform computation for some simple cryptographic functions (as SIM card can do in today’s GSM phones). The controlling unit then authenticated sensors (authenticator) and verified the authenticity of the sensor data forwarded by the monitoring software. When needed, the software can aggregate sensor data before sending it to the provider. The HMU adds message authentication codes to the messages sent to the provider and the device cannot confirm authenticity of the sensor data without the HMU. The HMU made the health-monitoring portable irrespective of the devices used and was easy to manage and hard to compromise.

In [46], authors argued that with healthcare systems being connected via IoT, smart medical devices are being exposed to numerous attacks [3]. This includes Trojan [62], malware (e.g., Medjack [63]), Sybil attacks using either hijacked IoMT [55] or single malicious nodes [64], DoS attacks [65], and man-in-the-middle (MITM) attacks [66]. To contain these attacks, authors proposed an automated threat analysis framework called SHChecker for Smart Healthcare Systems that uses a machine learning (ML)-based disease classification model (DCM) to deliver real-time treatment. SHChecker analyses the underlying decision-making model of Smart Healthcare Systems (SHS) by investigating the possible attacks that can be deployed with minimal alteration of sensor values. Therefore, to increase reliability, SHSs often employ data validation or anomaly detection systems. A clustering-based anomaly detection model (ADM) was added to the SHS due to its real-time detection capability. The ADM can learn the pattern of sensor measurement relationships by analyzing a huge quantity of data. The proposed SHChecker framework had the ability to assess potential attack vectors on SHS using ML algorithms, such as decision trees, logistic regression or could use an artificial neural network for classifying diseases and density-based spatial clustering of applications, with noise (DBSCAN) clustering algorithms to detect anomalies. The model’s efficacy was verified with several performance metrics using datasets from the University of Queensland Vital Signs and the synthetic dataset.

IoMT devices (e.g., medical sensors and actuators) are susceptible to various types of security threats, such as command injection flaws, insecure web interfaces, open ports, and insecure network services, thereby causing a significant risk to patient's privacy and safety [53]. These threats have given rise to several potential attacks against IoMT edge networks [13, 20], such as MITM attacks [66], spoofing attacks, traffic analysis attacks, masquerading attacks, and malware attacks. To overcome these attacks, authors modeled security threats for IoMT edge network using the Markov Chain and Common Vulnerability Scoring System (CVSS) that could help determine the probability of attacks for IoMT-based health systems. Furthermore, to protect IoMT edge networks against internal and external threats, a categorization of security countermeasures was presented for developing a proper and secure authentication and authorization access control mechanism.

In [54], authors presented an anomaly detection model for RHM utilizing IoMT and smart home devices that can detect malicious activities, such as theft of personal information, data breaches, and compromised medical devices [10]. The authors proposed the Hidden Markov Model (HMM)-based anomaly detection model that can analyze normal user behavior for RHM comprising of both smart health and smart home devices and identify any anomalous user behavior. The authors performed experiments by collecting use behavior data from multiple IoMT devices and home sensors and then trained the HMM model using these data. The proposed HMM-based anomaly detection model was considered to be over 98% accurate in identifying the anomalies in RHM systems.

Authors in [67] discussed security and privacy issues related to a widely used fitness tracking system, Fitbit. It was demonstrated that by reverse engineering the ANT protocol (ULP 2.4 GHz wireless radio networking protocol) and data communication, both active and passive attacks can be initiated on the system using off-the-shelf software modules. Attacks such as tracker injection (TI) attack, user account injection (UAI) attack, mule attack, and denial of service attack are most common on Fitbit devices. The various attack scenarios were analyzed to propose different types of possible defenses. A system termed ‘FitBite’ was built as a suite of tools that exploited these vulnerabilities to launch a wide range of attacks against Fitbit. Aside from eavesdropping, injection, and denial of service, there are several other attacks that can lead to financial gain and therefore are the most appealing to hackers. Another lightweight defense system called ‘FitLock’ was built to protect Fitbit, although it imposed a small overhead on memory. These systems and tools could be applicable to several other wearable and implantable healthcare systems.

## Enhancing RHM security using blockchain technology

The utilization of blockchain technology in the healthcare domain can be viewed across multiple use cases, but RHM is one of the most prominent areas as it affects the patients’ welfare and daily living. Security and privacy are the major challenges across the RHM applications, and blockchain has become the de-facto standard to overcome these challenges. This section describes the use of different types of blockchains, consensus algorithms, and smart contracts to address the challenges in RHM systems. Table 3 provides a summary.

**Table 3:** Comparative summary of existing architectures for RHM

Ref	Blockchain type	Smart contract	Implementation platform	Data storage	Cryptographic model
[68]	Private	Yes	Ethereum	Cloud	Unspecified
[69]	Private	Yes	Ethereum	Blockchain	Private/public key encryption
[70]	Public	Yes	Unspecified	Cloud	Blockchain
[71]	Private and public	Yes	Permissioned blockchain	Cloud	ARX encryption scheme, ring signatures and Diffie-Hellman key exchange
[72]	Pubic	Yes	PoW-based protocol (GHOSTDAG)	Blockchain	Satoshi's blockchain signatures
[73]	Unspecified	Yes	Ethereum	Blockchain	Unspecified
[74]	Public	Yes	PoW	IPFS storage(cloud)	PBFT consensus algorithm
[61]	Public	Yes	Ethereum	IPFS storage(cloud)	attribute-based encryption
[75]	Private	Yes	Ethereum	Blockchain	Unspecified
[76]	Unspecified	Yes	PBFT	Cloud and blockchain	symmetric key encryption
[77]	NA	No	No consensus	Cloud	Homomorphic encryption techniques and zero knowledge proofs
[78]	Permissioned	Yes	Hyperledger	Cloud and blockchain	Lightweight encryption and homomorphic encryption
[79]	Unspecified	Yes	Ethereum	Cloud and blockchain	Cryptographic parameter based encryption and decryption (CPBED) mode
[80]		Yes	Proof of authority (PoA)	Cloud	Digital signatures ( symmetric & asymmetric algorithms)
[81]	Unspecified	Yes	Ethereum and hyperledger	Blockchain and edge	Delegated proof-of-stake (DPoS)
[82]	Permissioned private	No	Unspecified	Cloud and blockchain	Zero knowledge proof

### Permissioned blockchain

An RHM system utilizing private blockchain networks and smart contracts was proposed in [68]. Being a private blockchain, only authorized viewers could read the block and only specified nodes could execute smart contracts to verify new blocks in the system. This limited the viewers to only authorized parties such as patients themselves, health care providers, and in some cases device manufacturers, thereby reducing access to information. The security was introduced through the concept of valid blocks that must contain signatures from a specified minimum number of members. No rogue nodes could insert false transactions into the chain as only pre-authorized verified nodes could be members. Cloud was used for storage due to the large quantity of data involved. The architecture predominantly exploited the advantages of the smart contract for security. The user interface (UI) is managed by a Decentralized Application (DApp) on the (smart) master devices, which were responsible for communicating with the smart contracts on the blockchain and managing user profiles.

An RHM system was proposed as part of [69] with three types of participants—hospitals, doctors, and patients registered within the hospital, with the encrypted registration information recorded by a smart contract [69]. In this architecture, hierarchical smart contracts were proposed for all kinds of entities and all the network peers were validated by the smart contracts to ensure security. Besides this, only the patient could authorize the doctor and hospital to check patient’s data. The authors did not propose any cloud storage or another database to record data but have used blockchain to store all data—with this approach, there is a possibility of making the blockchain expensive, data intensive, and slow. The article proposed a processing mechanism that intelligently filtered the data recorded by the smart devices from the patient’s body for data reduction. Any anomalous data were immediately written to the blockchain.

### Interoperability

Authors in [70] have discussed data integration as one of the major challenges for the use of IoMT in healthcare and provided a blockchain-based solution named ‘BlockIoT’. It was suggested that the data coming from multiple IoMT devices tend to be fragmented among health infrastructures, thereby preventing interoperability of medical data at the point of care. BlockIoT used blockchain technology to transfer previously inaccessible and centralized data from IoMT medical devices to EHR systems thus enabling greater insights to the healthcare providers. BlockIoT is an information transfer system combining a blockchain-based technology and uses APIs that enable interoperability between IoMT devices and EHR systems. It removed considerable overheads, such as development costs (required to modify existing medical device protocols) to transfer data between different parties and devices which use their own proprietary systems to manage data. The proposed system allowed individual medical device data to be securely transferred to providers without changing any firmware on medical devices or forcing companies to modify the existing protocols when creating medical devices. Another advantage of BlockIoT was the ability to present providers with essential data points by communicating and analyzing medical device data. For instance, healthcare providers or insurance agencies could use visual diagrams and algorithms to quickly analyze medical data and determine if a patient’s health was within normal limits or requires medical attention. BlockIoT was characterized by authors as a decentralized public blockchain system to store encrypted patient medical device data received from the API, which contained endpoints that received data published by a patient’s medical IoT device. As an example, a medical device can be a wearable heart monitor that could transmit a real-time heartbeat rate to a specific destination over a wired or wireless network. The article, however, does not provide details as to how the security will be managed and who will own the data. With decentralized architecture, hackers can enter into the blockchain network, thereby compromising data security [17].

### Blockchain scalability

Authors in [71] reviewed the regulations around confidentiality, collection, storage, and exchange of patient’s data and their compliance toward standards such as GDPR and HIPPA. As an example, with regards to integrity, article 5(d) of the GDPR states that the data of patients should be kept accurate and up to date. Based on article 32 of the GDPR, security measures should be adapted, so that patients could get access to their data in time. The authors then reviewed the blockchain-IoMT systems and presented approaches to improve the security, privacy, and scalability of blockchain technology for RHM systems using a combination of cloud and blockchain. Most IoMT systems are cloud based which is not well suited for RHM due to the large, distributed scale of IoMT networks and single point of failures [10, 15]. In addition, security and privacy are the major challenges in IoMT as devices are resource-limited and cannot use complex security algorithms. Furthermore, the authors also discussed the limitations of using conventional cryptographic primitives and access control models to address security and privacy issues in the cloud-based environment [25]. To overcome these challenges, authors in [71] have discussed several approaches to improve the scalability of blockchain technology which includes on-chain and off-chain techniques, based on which they have given recommendations and directions to design a scalable blockchain-based IoT system. They recommended that a designer should consider the well-known trilemma along with the various dimensions of a scalable blockchain system to prevent the sacrifice of security and decentralization as well. Furthermore, they recommended that a type of lightweight symmetric algorithm named ARX is used to provide confidentiality for patient’s data and the model also supported authentication by means of a digital signature.

In [72], a remote monitoring device was used to monitor and transmit health data to smart contracts, a smartphone with Internet connectivity, and an RHM app to receive data. Patient data were sent to the central server which hosted the blockchain and health providers could use the blockchain to retrieve patient’s information. As the blockchain had limited scalability, authors introduced a PoW-based protocol named GHOSTDAG, that used Satoshi's blockchain to provide high throughput while also avoiding the security and scalability issues. Satoshi Nakamoto invented the Blockchain in 2008 to serve as the public transaction ledger of the cryptocurrency bitcoin [26]. The solution was built on a private blockchain network which uses the GHOSTDAG protocol. Using a private blockchain, authors have created a system using smart contracts to analyze the patient’s health data. For an abnormal reading, the smart contract issued an alert, which was also recorded to the public blockchain. The authors state that this solution resolved the privacy and security vulnerabilities associated with RHM and also the limited scalability problem of Satoshi's original blockchain.

### Data volume and security using a combination of blockchain and cloud

Cloud has become mainstream and caters to a variety of solutions to store patient’s records [73]. Such solutions are, however, affected by security issues, low response time, and the low availability of the system. To overcome these challenges, an intelligent IoT-based distributed framework was proposed as part of [73] to deploy remote healthcare services. In the proposed model, various entities of the system were interconnected using IoT devices. A distributed database management system was used to enable secure and fast data availability to the patients and health care workers. The proposed framework comprised of four layers: hospitals, remote IoT-integrated medical nodes, distributed medical records, and AI-based smart contracts. The hospital layer acted as an information warehouse where complete patient’s healthcare records were stored. The distributed medical records layer was integrated with the hospital layer and medical records stored in the hospital layer were distributed across the different nodes to make them secure using a blockchain network. The AI-based smart contract layer was merged between the hospital and distributed medical records’ layer, IoT nodes, and hospital layer. The remote IoT-integrated medical nodes were then used to sense the various health parameters of the patients and to transfer the sensed data securely to blockchain protected distributed databases. The proposed model comprised of intelligent analysis of the clinical records fetched from distributed database management systems secured with blockchain using smart contracts.

Privacy leakage and single point of failures were identified as the two major challenges for RHM [10, 11] as users’ health data are generally stored in centralized third-party servers, such as the hospital database or cloud [74]. The authors [74] proposed a public blockchain-based smart healthcare system called `Healthchain' that allowed patients to store their large-scale health data securely and with a privacy-preserving scheme based on blockchain technology, where health data were encrypted to conduct fine-grained access control. In particular, users could revoke or add authorized doctors to their health data by leveraging user transactions for key management. Furthermore, by introducing Healthchain, both IoT data and doctor diagnosis could not be deleted or tampered with, to avoid medical disputes. Using this model, authors aimed to ensure that no adversary could get access to user's sensitive health data. The authors also tried to define a mechanism to audit tampering of doctors' diagnosis (if any) as well as providing an option for patients to deny a doctor’s right to access to their health data. The security analysis was performed to ensure performance evaluation of the design goals. The authors did not, however, describe how to share and evaluate the EMRs transmitting processes.

In [61], authors have introduced a novel EHRs sharing architecture which gathered information from wearable devices with the help of blockchain and InterPlanetary File System (IPFS) for a healthcare system. To enhance the security of EHRs’ sharing, they used smart contracts that provided a trustworthy access control mechanism. The suggested system was based on a mobile cloud architecture in which the patient data were collected from a set of local gateways. The data were then stored on a cloud for the purpose of data sharing with healthcare providers. The EHRs also gathered data from wearable body sensors with the help of a mobile application on patient’s smartphones. A cloud blockchain network was also proposed for the sake of data sharing. This blockchain was based on Ethereum and its main components constituted an EHR Manager that controlled the transactions of all the users on the blockchain network, thus playing a crucial role in the data sharing framework. An admin component could manage operations and transactions on the cloud and was responsible for adding, revoking, and changing access permissions. Smart contracts were used as the core software in the proposed healthcare platform to define access control. As part of decentralized storage, IPFS provided a decentralized peer-to-peer file system to allow a file sharing platform in the blockchain network. The design goals of the proposed architecture were to ensure that only authorized users were authenticated by the system to access EHRs.

In article [76], authors pointed out that security experts and regulators have strongly challenged storing data on the cloud due to insufficient data privacy and data controls [13]. Due to the recent increase in cases of security and surveillance breaches compromising patient’s privacy, many questions have been raised on the conventional models where third parties gather and store immense amounts of patients' healthcare data on the cloud [16]. To mitigate this concern, a blockchain-based protocol was proposed for e-health applications that does not require trust in a third party and provides an efficient privacy-preserving access control mechanism. As IoMT devices have resource constraints, authors have not used the conventional methods for consensus operations in blockchain such as proof of work (PoW), due to its inherent challenges [11]. Instead, practical byzantine fault tolerance (PBFT) was used as a consensus method. PBFT requires multiple rounds of voting by all the nodes on the network which makes it more secure. PBFT can help reduce network costs, i.e., bandwidth, number of processors, and energy for consensus operations. From a data storage perspective, authors have proposed the storage of the data on the cloud (off-chain) instead of storing all data on the blockchain ledger. In addition, authors proposed the use of a symmetric key encryption scheme to secure the data and address data security on cloud.

Aggregating heterogeneous data from different types of sensors and other sources poses significant challenges for RHM. The IoMT devices can generate voluminous data characterized as ‘big data’ which require processing times that range from batch, to pseudo-real, to real-time processing of various types of data (e.g., text, audio, video, etc.) which was one of the biggest challenges. To overcome this challenge, authors in [77] proposed an agile software infrastructure that embraced cloud and fog computing, blockchain, Tor browser (also known as the onion router) and message brokers for flexible, cost efficient, secure and privacy-preserving deployment of IoT for smart healthcare applications and related services. The privacy of patient records and other confidential and sensitive healthcare data was ensured using Tor browser in tandem with machine-to-machine (M2M) message protocols, such as the MQTT to protect the privacy of online users and data. However, since Tor on cloud could introduce delay and unpredictability, authors proposed that it should be employed between fog nodes and the cloud. Fog nodes are more localized and can meet the stringent real-time requirements [12], in contrast to Tor in the cloud. The authors used blockchain to secure sensitive data by tracking and authorizing access to confidential medical records and data received from RHM devices and also provided the option of using a private or public blockchain.

The Industrial Internet of Things (IIoT) connects several devices to the Internet, but many of these devices cannot ensure adequate security and privacy [78]. One of the main reasons is that these devices were never designed to connect to the internet. To address this challenge, authors in [78] developed a security framework that is based on blockchain and deep-learning techniques. First, a blockchain schema was designed where each entity that wants to participate in the network were registered. This meant that using smart contract-based enhanced proof of work, the participants were verified, thereby addressing the first level of security and privacy checks. Second, the framework was developed to use deep-learning techniques to further enhance security and privacy. The proposed model learns from the behavior and integration of users to provide a decentralized nature and privacy preservation approach. The deep-learning scheme uses a Variational Auto-Encoder (VAE) technique for privacy and bidirectional long short-term memory (BiLSTM) for intrusion detection. The proposed simulation results were compared with the benchmark models, and it was confirmed that the proposed framework outperformed the existing system.

On one side, healthcare is seeing a massive adoption of digital technologies with the emergence of IoMT systems, but, on the other hand, it brings in several security risks that need to be handled effectively to preserve the trust among all engaged stakeholders [79]. Traditional models have tried to address security and privacy using several authentications and data preservation schemes; however, it seems these have never been fully addressed. To bridge these gaps, authors in [79] have proposed a lightweight authentication and data preservation scheme for IoT-based cyber-physical systems (CPS) utilizing deep-learning (DL) to facilitate decentralized authentication among legal devices. The framework consists of three layers—a device layer constituting of IoT-based industrial tracking systems and implantable medical devices, an edge layer constituting of edge-computing servers, industrial computers, and data analysis servers and finally a blockchain powered cloud layer where various cloud suppliers and data centers are included. Authors also proposed blockchain-based fog consensus protocol (BFCP) to tackle the problem of the privacy of sensitive healthcare data. The protocol employed a group of nodes in a federated capacity to provide strong security to the blockchain. The experiments demonstrated an accuracy of 94.2% for simple EMR and 93.3% for encrypted EMR, thereby serving as a tool to classify encrypted data by non-trustworthy third parties without disclosing confidentiality.

### Data sharing

Authors in [80] have applied blockchain to a health application network where patient’s health data can be used to generate an alert to authenticated healthcare providers in a secure and private manner. The proposed solution considered a typical RHM system where patients are equipped with wearable healthcare IoT devices to collect health data, such as heartbeats, distance walked, or sleep conditions. The patient in the system had the final authority to grant or deny data access to any external parties, such as healthcare providers or doctors. Whenever a patient needs treatment, they could share their health data with appropriate healthcare providers and revoke access after the treatment is over. The proposed architecture used proof of authority (PoA) and relied on digital signatures of nodes for authentication. To decrease network overhead and delay, the architecture divided the blockchain into clusters through an overlay network. Instead of using a single blockchain, clusters were used to avoid delays (due to low bandwidth) in the network and to reduce overhead. Each cluster constituted a group of several nodes, and one node was treated as a cluster head (CH). Each cluster elects a CH and each of them maintained a unique public key which is known by all other CHs. In this manner, new blocks were generated for the chain and the CH can directly authorize the block generator. As per the authors, the proposed blockchain-based IoT model handles most privacy and security threats while considering the resource constraints of many IoT devices.

In [75], the authors have emphasized the concerns raised by the health service providers and insurance companies on the weak verification models being adopted on the data generated by wearable sensors. It was argued that without a strong verification model, attackers could attempt to manipulate the data transmitted from a health sensor such as an insulin pump which can pose a huge risk to patients as there is a danger of a lethal dose of medicine being received. To address these security and privacy concerns, a verification framework using blockchain was created whereby various authorities can request a verification service for the local network data of a target IoT device. The proposed solution leveraged Ethereum public blockchain and smart contracts as a distributed platform to realize an on-demand verification scheme. The proposed scheme was evaluated using real Wi-Fi session traces collected from a five-story building with more than 30 access points, representative of a hospital. The model enabled healthcare authorities to verify data transmitted from a typical wearable device with the verification error of the order 0.01% [61].

Intelligent-health (I-Health) systems were developed to aggregate diverse e-health entities in a unique national healthcare system by enabling swift, secure exchange, and storage of medical data [81]. In particular, the solution was designed as an automated patient monitoring scheme, at the edge, which enabled the prompt discovery, remote monitoring, and fast emergency response for critical medical events, such as emerging epidemics using a combination of IoT and blockchain. A blockchain optimization model was proposed as part of the system, so that the acquired data from various entities were treated based on their urgency and security levels. For example, urgent data should be given the highest priority and dealt with using a restricted blockchain, i.e., with a minimum number of validators. On the contrary, for low priority data requiring a high security level, a fully restricted blockchain was used. The authors emphasized that the processing and sharing of data at the edge with a blockchain network can help in managing urgent outbreaks, such as COVID-19. The solution could work with either a public or private blockchain.

### Protection of edge devices

In [82], a lightweight permissioned blockchain mechanism was discussed for the RHM system at the edge. Edge computing is a computing model to extend the cloud to the network edge to enable efficient data access, computation, networking, and storage [12, 21]. The authors discussed a generalized architecture for transmitting the in-house healthcare monitoring data to the cloud by leveraging edge nodes. A decoupled blockchain-based model was proposed for data privacy and data security preservation in the in-house healthcare monitoring ecosystem. An incremental tensor train decomposition model was discussed to store the healthcare data in the cloud and prevent data duplication. The model had three layers: IoT-healthcare, the edge-blockchain, and cloud for storage. The IoT-healthcare layer consisted of IoT health devices that continuously read and sent data to edge servers. The edge-blockchain layer comprised of edge servers throughout the region stored only in the header and block ledger, whereas the entire data was stored in the cloud layer. Nearby edge devices in the first layer create decoupled blocks in blockchain to transmit data from sensors to edge nodes, and the edge nodes in the second layer transmitted and stored the data on the cloud. A patient’s block in the chain could be accessed by an ID and a public key which ensured the security of data. The incremental tensor train decomposition scheme for dimensionality helps to remove the unwanted attributes or dimensions of the high-dimensional data generated by healthcare sensors, thereby extracting a compact version of the data which on one hand reduces storage space and on the other hand relieves the load on the network during transmission. This scheme was considered as one of the modern ways in which data can be compressed and transmitted.

## Service scenario and open research challenges

### Service scenario

We describe a typical remote patient monitoring system service scenario that can alert a consulting doctor when a patient with a history of heart attacks encounters abnormal heart readings, i.e., heart readings > 140 beats per minute. Atrial fibrillation is a heart condition that causes an irregular and often abnormally fast heart rate and is one of the major reasons for heart attacks [83]. This forms the basis for our service scenario.

**Fig. 4:**
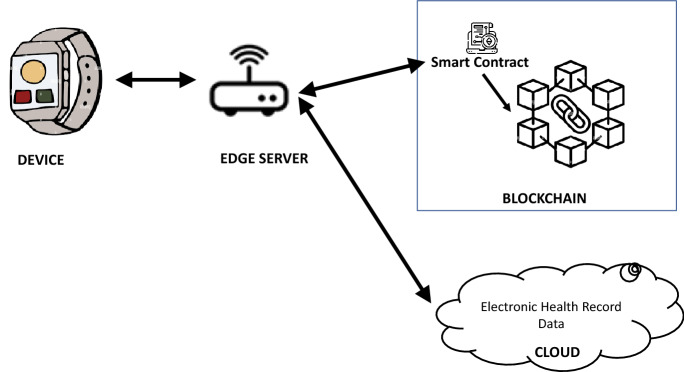
Remote patient monitoring service scenario

As depicted in Fig. 4, the device layer sends data from the heart rate monitoring devices attached to the human body.

Aside from data delivered by the wearable heart monitoring device, a remote monitoring system needs patient electronic health record data which consists of their past medical history (including past occurrences of heart attacks), which is generally stored in the cloud. These data combined with patient real-time data from the sensor forms the basis for the RHM system to detect the heart attack risk.

Data received from the heart rate monitoring device are stored on the blockchain after filtering all normal readings (heartbeats < 140) at the device layer itself. This is performed by an edge server deployed near the devices which is capable of connecting with the heart rate monitoring device. As an example, the edge server is programmed to send only abnormal heart rate > 140 beats per minute to the blockchain layer, whereas the reminder of the data is sent to the cloud for storage. Once a series of abnormal heart rates are detected, IoT devices invoke smart contracts and log all events on the blockchain.

The smart contracts support real-time patient monitoring for heart rate and can be programmed to send notifications to the doctor in case there are multiple occurrences of heartbeat > 140 beats per minute, in parallel to storing all abnormal heart readings on the blockchain. This can facilitate medical intervention for the patient. Using smart contracts, the system would resolve many security vulnerabilities associated with remote patient monitoring and automate the delivery of notifications to all involved parties in an HIPAA compliant manner.

### Open research challenges

The utilization of blockchain technology for RHM use cases is considered as one of the most efficient models due to the inherent nature of the technology, deemed to be tamper-proof, distributed, and incorruptible, thereby facilitating the access of patient data in a secure way.

**Fig. 5:**
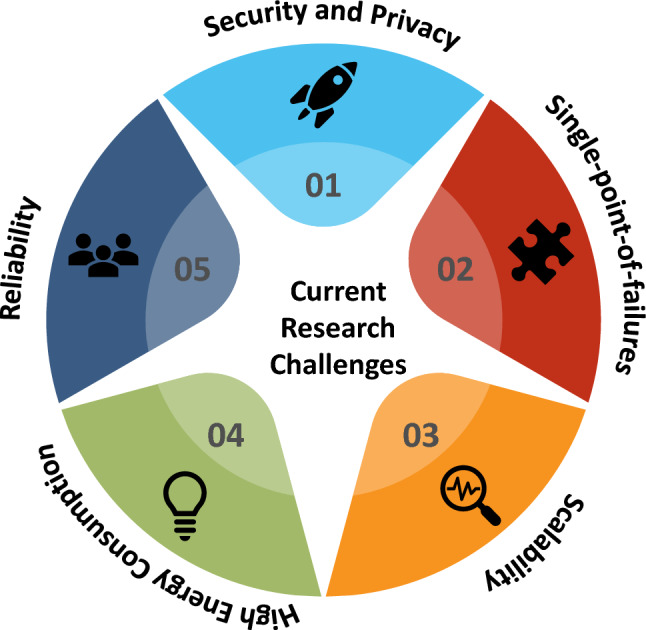
Blockchain and IoMT challenges

Based on the review, we can deduce that there are still several open research gaps, as depicted in Fig. 5, that still need thorough investigation while integrating blockchain with RHM use cases. These challenges can form the basis for future research direction.

#### Security and privacy

Several papers have highlighted the risks of security and privacy for RHM use cases and have suggested using blockchain to address these issues [9–11, 13, 17, 24, 28].

The vulnerability of blockchain endpoints is a vital concern in blockchain security as investigated in this review. It is clear that this area has not been fully explored until now. To understand the vulnerability of blockchain endpoints, for example, Bitcoin trading could result in a large amount of Bitcoin stored in a virtual savings account. The actual blocks can be secured against hackers using solutions provided as part of [10, 17, 25, 28], but there is little focus on safety of the wallet accounts. Furthermore, many third-party vendors also facilitate blockchain transactions. Some of these third-party vendors include blockchain payment platforms, payment processors, and smart contracts. Such third-party blockchain vendors can increase vulnerability to hacking due to weaker security in apps and websites.

#### Single point of failures

There are several papers which suggest using private blockchains for RHM use cases [68, 71, 75, 82]. However, one of the major challenges with private blockchains is the single-point-of-failure.

A private blockchain follows a centralized architecture, which means that it is controlled by one entity or enterprise. The challenge with this architecture is that there is a central coordination system within an enterprise, in which each node is connected to the systems as part of the blockchain and the information gets shared between them. In this case, since the network is owned by just one entity or enterprise—there is a problem if central coordination systems fail, since all of these individual nodes and systems will get disconnected and the network may shut down leading to a single-point-of-failure [15], which can potentially disrupt the entire network [16, 17]. This is an area which needs further investigation to find a potential solution.

#### Scalability

Scalability is one of the biggest drawbacks of blockchain technology as blockchain cannot be scaled up due to the fixed size of the block for storing information. RHM use cases generates enormous amounts of data and it is practically impossible to store this data on the blockchain. This has not been thoroughly investigated in the research literature. Although there were ‘on-chain’ and ‘off-chain’ techniques proposed as part of [71] to improve the scalability of blockchain technology, with only the transactions stored on the blockchain (on-chain) and rest of the data stored in a database (off-chain), these kinds of models are still far from perfect.

#### High-energy consumption

Despite digital currencies providing considerable potential transactional and financial benefits, the design of blockchain and blockchain mining consumes vast amount of electricity that could be equivalent to powering the whole of Denmark [84]. Even the processes involved in a single Bitcoin transaction could provide electricity to a British home for a month [85]. Although it has been argued that such blanket statements about blockchain energy consumption are exaggerated and need to be reviewed with care [86], solutions to tackle this perceived challenge are far from being materialized.

#### Reliability

Blockchain is known to be highly reliable [71, 73, 75, 80] which is one of the reasons why it has become so popular. However, one specific gap in blockchain which has not been addressed so far in the area of reliability is oracles. Oracles are off-chain components that reside outside the blockchain systems which could be points of failure. The reliability of blockchain oracles needs to be thoroughly investigated and addressed to make the network fully compliant and reliable.

## Discussion

There is a continuous threat to the integrity of sensitive healthcare data if it is not secured and managed properly [15]. This challenge is further aggravated for RHM use cases, since data are vulnerable to cyber threats during communications. Best practices to effectively manage and store data are vital to address these security and privacy issues. Blockchain is a technology that is receiving widespread acceptance and adds a lot of value to overcome these challenges [23].

Based on this study, it is evident that a substantial portion of RHM-related articles emphasize the application of blockchain to overcome security and privacy concerns for RHM. Several authors have stated that patient’s data are traditionally stored separately across different databases with little or no interoperability. This leaves the control of the health data mostly in the hands of the service providers and limits the collaborative sharing of such data among healthcare stakeholders.

By applying blockchain to the management of RHM, patients can be in control of their own health data and are able to decide how the data are used while addressing concerns on privacy and security.

Specifically, with regards to the security and privacy of public blockchain-based healthcare applications, it is evident that that despite the encryption techniques employed, it is still possible to reveal the identity of a patient and corresponding data in a public blockchain by linking together metadata associated with said patient [69]. In addition, there are potential risks of security breaches that could arise from malicious attacks to the healthcare public blockchains by criminal organizations that could compromise the privacy of patients [14, 27, 71, 74, 87]. There have been several cases of reported attacks on the public blockchain networks that power different cryptocurrencies. This makes public blockchain an area of concern—yet they are still often recommended for RHM use cases. With respect to private blockchain-based healthcare applications, there are models that have been discussed in the literature that can address the security and privacy issues such as in [68, 71, 75, 82]. The prototypes in these literatures have demonstrated that they are suitable for complex uses cases as well, which looks promising.

An important research gap from a technology perspective which seems to be left unaddressed in the literature for private blockchain is in the area of data availability i.e., single point of failures. By default, it is considered in most literatures that private blockchains due to the distributed nature of the nodes will not have a single-point-of-failure, so the network is less likely to experience downtime which is not correct and needs a solution. Aside from this single-point-of-failure challenge, there are other gaps in blockchains that are yet to be addressed in the areas of security and privacy, reliability, scalability, and energy consumption which need further investigation.

Finally, with respect to the use cases that have addressed the area of RHM systems, this study reveals that almost all the articles consider RHM for routine health checks, such as blood pressure or glucose level [20]. However, RHM using a combination of IoMT and blockchain can reinvent the health sector with preventive healthcare [2] for life-threatening diseases, such as heart attacks and cancer.

## Conclusion

Blockchain-based solutions in RHM systems are becoming essential due to an increasing number of security threats. The blockchain technology provides significant advantages over other solutions in addressing the security and integrity, latency, and reliability challenges due to its salient features of decentralization, immutability, transparency, and interoperability. The utilization of blockchain for RHM use cases has many benefits for the patients, healthcare professionals, and related stakeholders. Patient’s data can thus be acquired, shared, and analyzed for generating timely recommendations and interventions while ensuring security and privacy is maintained.

In this review paper, we have conducted a comprehensive review of the blockchain-based RHM systems across both private and public blockchains. It is clear by analyzing these studies that public blockchains still have several limitations, but in comparison, private blockchain models can overcome security and privacy concerns. The fact that private blockchains have a single-point-of-failure also needs to be addressed.

## Data Availability

The material used and/or analyzed during the current study are referenced/cited appropriately in the paper.
